# Ventilation-Based Decellularization System of the Lung

**DOI:** 10.1089/biores.2016.0012

**Published:** 2016-05-01

**Authors:** Tomoshi Tsuchiya, Julio Mendez, Elizabeth A. Calle, Go Hatachi, Ryoichiro Doi, Liping Zhao, Takashi Suematsu, Takeshi Nagayasu, Laura E. Niklason

**Affiliations:** ^1^ Departments of Anesthesia and Biomedical Engineering, Yale University, New Haven, Connecticut.; ^2^ Division of Surgical Oncology, Department of Translational Medical Sciences, Nagasaki University Graduate School of Biomedical Sciences, Nagasaki, Japan.; ^3^ Division of Electron Microscopy, Nagasaki University Graduate School of Biomedical Sciences, Nagasaki, Japan.

**Keywords:** decellularization, extracellular matrix, lung, mandatory ventilation, tissue engineering

## Abstract

The demand for donated organs greatly exceeds the availability. Alternatives to organ donation, such as laboratory-engineered organs, are therefore being developed. One approach is to decellularize the organ and reseed it with selected cells, ideally from the organ recipient. Organ decellularization has typically been attempted by the administration of detergents into vessels such as the portal vein in the liver. However, in the case of the lung, the airway provides another potential administration route, because it has a wide contact area between cells and detergents in the tracheal tree and alveoli. In the present study, we introduce a novel ventilation-based decellularization system for the lung and compare its efficacy to ordinary decellularization systems administering detergent through the pulmonary artery. Rat lungs were decellularized using 500 mL of 3-[(3-cholamidopropyl) dimethylammonio]-1-Propanesulfonate (CHAPS) decellularization solution administrated through the pulmonary artery (vessel group) or through the trachea (airway group). The vessel group was infused CHAPS solution using a gravitational pressure head of 20 cmH_2_O. The airway group was infused with the detergent using negative pressure and positive end-expiratory pressure, for a volume 10cc with each inspiration in a bioreactor. Pathological and immunohistochemical findings indicated that components of the extracellular matrix (ECM), including proteoglycans, elastic fibers, fibronectin, and laminin, were more decreased in the airway group than in the vessel group. Western blot analysis showed that MHC class I antigen and β-actin were not detected in both decellularized groups. A collagen assay showed that collagen was 70% preserved in both groups compared to native lung. Glycosaminoglycan (GAG) and DNA assays showed that GAG and DNA contents were strongly diminished in both decellularized groups, but those contents were smaller in the airway group than in the vessel group. Accordingly, the alveolar wall was thinner on electron microscopy, and DNA remnants were not observed in the airway group. Infusion of red blood cells indicated that capillary walls were preserved without blood leakage in both groups. In conclusion, we describe a novel approach for decellularization through the airway that represents a more stringent method for both DNA and ECM removal, with capillary wall preservation.

## Introduction

The generation of laboratory-engineered functional organs would be a major advancement in meeting the demand for organs for transplantation. Biologic scaffolds derived from decellularized tissues have been successfully used in human clinical applications, including heart valves, blood vessels, trachea, and small intestine.^1–3^ These seminal studies and our own previous reports show that, even if imperfectly, recellularized solid organs can perform organ-specific functions, which indicate the potential for clinical use of engineered solid organs in the future.^4–7^

The main method of decellularization using detergent is to incubate tissues in detergents such as 3-[(3-cholamidopropyl) dimethylammonio]-1-Propanesulfonate (CHAPS), sodium dodecyl sulfate, or sodium deoxycholate. For solid organs, including heart, liver, or lung, the detergents are typically administrated through vessels such as the coronary artery, portal vein, or pulmonary artery.

The lung is the respiratory organ, in which gas exchange is performed between blood and alveolar air through capillary endothelium and alveolar epithelium. Accordingly, the organ is dominated by two systems comprising vascular tissue and airway or respiratory tissue. The area of the respiratory field is extremely wide (in the human, 60–70 m^2^), suggesting that decellularization through the airway might be more effective than that through the vasculature for completely removing cellular and nuclear components. We studied whether mechanical ventilation of the respiratory tissue might facilitate the perfusion and drainage of the entire organ with decellularization solutions.

Mechanical ventilation is categorized into two types as follows: conventional positive pressure-based ventilation and negative pressure-based ventilation using an “iron lung.”^8^ Positive pressure ventilation opens airspaces from the central region, but the conducting airways are often obstructed or the airspaces cannot be expanded, because of local surface tension, especially in the periphery of the lung. Thus, applying positive pressure may not recruit atelectatic (degassed) units and may overdistend other already inflated units. In contrast, negative pressure ventilation starts by opening airspaces from the peripheral region and is demonstrated to have an efficacy for decreasing atelectatic units.^9^ Therefore, in the present study, we used a negative pressure ventilation system in a bioreactor, similar to the concept of the “iron lung.”

Clinically, positive end-expiratory pressure (PEEP) is often applied to ventilation to avoid the collapse of alveoli. We decided to include PEEP in our decellularization process to promote uniform results throughout the lung. Using the original equipment, we designed for recellularization of lungs; the new decellularization system was altered to incorporate PEEP by redesigning the piping of the chamber. Consequently, we tested whether the new system produces uniform decellularization, especially at the periphery of the lung. We also assessed the quality of the extracellular matrix (ECM) of the decellularized lung scaffold.

The purpose of the present study is to compare the efficacy of decellularization of the lung by administering detergent through the airway using a simple negative pressure bioreactor system. To evaluate matrix damage and decellularization efficacy, including removal of cytoplasmic and nuclear components, we analyzed histological and matrix components in rat lung tissues following decellularization through the trachea.

## Materials and Methods

### Organ harvest and decellularization

**FIG. 1.**
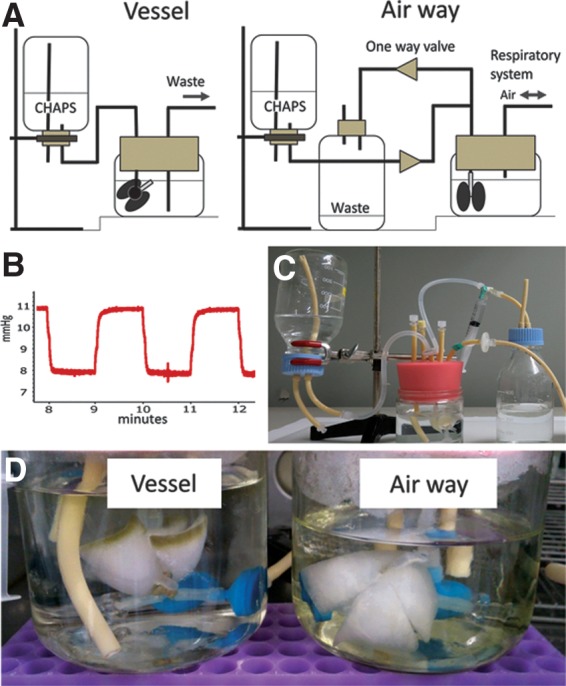
Schematic illustration of the system of the airway decellularization **(A).** Internal pressure of the bioreactor in the ventilation-based decellularization system **(B)**. Airway-mediated decellularization system **(C).** Gross findings of decellularized lungs through vessel or airway **(D)**. Note, decellularization is not completed at the peripheral region of the vessel group.

Lung tissue was obtained from young, adult (3-month-old) male Sprague-Dawley rats (SD rats, Charles River, Wilmington, MA). All animal experimental work was performed with the approval from the Yale University Institutional Animal Care and Use Committee. All animal care complied with the Guide for the Care and Use of Laboratory Animals. Organ harvest was performed as previously described.^6^ The harvested organ was placed in a 500 mL glass jar containing phosphate buffered saline (PBS) with heparin (250 U/kg). The cannulae were connected to a vessel loop and airway loop inserted through a silicone stopper forming the basis of the bioreactor (Fig. 1A, C). All bioreactor components were obtained from Cole-Parmer (Vernon Hills, IL).

The harvested lungs were divided into three groups of five replicates consisting of (a) vessel group (detergent administered through the pulmonary artery); (b) airway group (detergent administered through the airway); and (c) native control group (tissue harvested without perfusion with CHAPS solution). In the vessel group, the lung was decellularized by 500 mL CHAPS through the pulmonary artery (8 mM CHAPS, 1 M NaCl, 25 mM EDTA, and pH = 12 in PBS). The arterial decellularization procedure was similar to a technique described previously.^6^ Briefly, lungs were cannulated at the pulmonary artery trunk and the vasculature perfused with decellularization fluids at 37°C with pressures kept below 20 cmH_2_O. The perfusion time was ∼2 and a half hours. After decellularization, tissues were extensively rinsed using vascular perfusion with 2500 mL of PBS.

In the airway group, lungs were cannulated at the trachea and perfused with CHAPS solution at 37°C through the trachea. Ventilation was performed in the closed bottle bioreactor described above, using negative and positive pressure at 0.5 breaths per minute, using a 20 mL syringe on a programmable infusion pump (Cole-Parmer). The tidal volume was 10 mL. Ten cmH_2_0 pressure was continuously added using fluid level gaps of CHAPS solution (Fig. 1A). As the syringe is pulled and pushed by the pump, the pressure inside of the bioreactor alternated between 8 and 11 mmHg (∼11 and ∼15 cmH_2_O) to produce inhalation and exhalation (Fig. 1B). As a result, a total of 500 mL of decellularization fluid flow from the stock reagent bottle, into the lungs, and are eliminated into the waste bottle using one-way valves (Fig. 1A). It took approximately 2 h for decellularization. After the decellularization procedure, tissues were extensively rinsed with 2500 mL of PBS at one breath/minute using the same system. The pressure inside of the bottle was measured by a PowerLab2/26 (ADInstruments, Dunedin, New Zealand).

### Histological analysis and immunohistochemistry

Tissues were formalin fixed, paraffin embedded, and sectioned at 5 μm thickness. Analysis was performed with standard hematoxylin and eosin staining. Elastic Van Gieson (EVG) was used for elastin staining. Alcian blue was used to detect proteoglycans and glycosaminoglycan (GAG) chains. DAPI (4′, 6-diamidino-2-phenylindole) was used for DNA detection. For immunohistochemistry, antigen retrieval was performed with 20 ng/mL proteinase K in TE buffer (pH 8.0), at 37°C for 10 min. Sections were then blocked with 5% bovine serum albumin and 0.75% glycine in PBS for 1 h at room temperature. Primary antibodies of monoclonal anti-rat fibronectin (ab6328; Abcam, Cambridge, United Kingdom) and polyclonal anti-rat laminin (ab11575; Abcam) were applied at a concentration of 1:100 in blocking buffer overnight at 4°C, followed by secondary antibodies at 1:500 dilution for 1 h at room temperature. Primary antibody of monoclonal anti-rat β-actin (ab8226; Abcam) was applied at a concentration of 1:200 in blocking buffer. Secondary antibodies used were mouse anti-rat antibody at 1:500 (Invitrogen, Carlsbad, CA). Slides were mounted using DAPI-containing mounting media (Vector Laboratories, Burlingame, CA).

### Western blotting

Tissues were digested in RIPA buffer (Boston BioProducts, Ashland, MA) with freshly added protease inhibitors (Sigma) and homogenized at 15,000 rpm for 30 sec. After incubation for 1 h at 4°C, insoluble particles were removed by centrifugation at 14,000 g for 25 min. Protein concentration was quantified using the Bradford assay and then boiled in Laemmli's reducing buffer (Boston BioProducts) for 25 min at 65°C. Protein was electrophoresed on polyacrylamide gels, transferred to a nitrocellulose membrane, and blocked for 2 h in 5% nonfat dry milk (NFDM) in TBS with 0.05% Tween-20 (TBS-T). Primary antibodies of monoclonal anti-rat β-actin at a dilution of 1:2000 (Sigma) and monoclonal anti-rat MHC class I (major histocompatibility complex) antibody of RT1A at a dilution of 1:500 (BD Pharmingen, BD Biosciences, CA) were applied overnight in 1.5% NFDM in TBS-T, followed by horseradish peroxidase–conjugated goat secondary antibodies (Santa Cruz Biotechnology, Santa Cruz, CA) for 1 h at room temperature at a dilution of 1:2000. Protein was detected using substrate from SuperSignal West Pico.

### Collagen assay

Collagen was quantified with a colorimetric assay to detect OH-proline using a modification of Grant's [1964] method. Lung samples were lyophilized and weighed, then incubated in papain (10 U/mL; 25 mg/mL) at 60°C overnight (Sigma). Papain-digested samples were incubated in 6 N HCl at 115°C for 18 h, neutralized, oxidized with Chloramine-T, and reacted with p-dimethylaminobenzaldehyde. Absorbance was measured at a wavelength of 550 nm, and a 1:10 w/w ratio of hydroxyproline to collagen was used to calculate the collagen content of the tissue.

### Sulfated GAG and DNA assay

Sulfated GAGs were quantified using the Blyscan GAG Assay Kit (Biocolor). DNA content of tissues was quantified using the Quant-iT PicoGreen dsDNA Assay Kit (Invitrogen, Eugene, OR), following the manufacturer's instructions.

### Scanning electron microscopy

Samples were fixed using 2% glutaraldehyde and 2.5% paraformaldehyde in 0.1M cacodylate buffer (EMD Biosciences, Gibbstown, NJ) for 2 h at room temperature, then rinsed in cacodylate buffer, sliced, and dehydrated through an ethanol gradient. Samples were further dehydrated in hexamethyldisilazane for 10 min, dried overnight, then sputter coated with gold, and analyzed using a JOEL JXA-8600.

### Red blood cell administration test

To assess basement membrane integrity, we used human red blood cells of 8 μm in diameter. Ten millimeter of diluted human packed red blood cells in PBS buffer (RBC; Hb 1.9 g/dL) was gravity infused into the decellularized lungs through the pulmonary arteries using a 10 mL syringe. After confirmation of the drainage from the pulmonary vein, decellularized lungs were fixed and analyzed by Masson's trichrome stain.

## Results

### Gross appearance

The gross appearance of both groups of decellularized lungs looked similar, white or faintly colored, at the central portions of the organ. There was some brown residue observed at the periphery in the vessel group (Fig. 1D).

### Histological findings

**FIG. 2.**
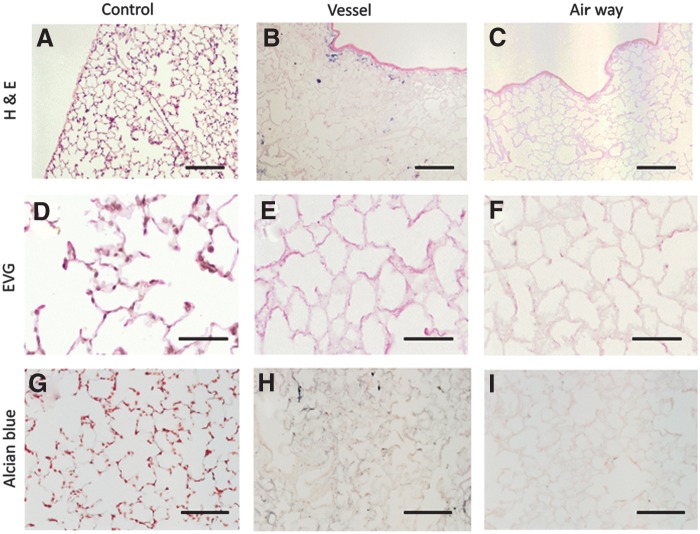
Upper panel shows hematoxylin and eosin staining of decellularized lungs. No intact nuclei are observed in decellularized lungs **(B, C)** compared to native lung **(A)**. Middle panel shows Elastic Van Gieson (EVG) staining of decellularized lungs **(E, F)**. Elastin shows deep wavy staining at elastic fibers in native lung **(D)**. The staining is weaker in vessel-mediated decellularized lung and airway-mediated decellularized lung **(E, F).** Lower panel shows alcian blue staining of decellularized lung **(H, I).** Blue staining, representing glycosaminoglycan (GAG), is clearly observed in native lung **(G).** The staining is weaker in vessel-mediated decellularized lung and airway-mediated decellularized lung **(H, I)**. Scale bar = 200 μm **(A–C)**. Scale bar = 50 μm **(D–F)**. Scale bar = 100 μm **(G–I)**.

When rat lungs were subjected to detergent decellularization by either the airway or the vasculature, the decellularized scaffolds were completely devoid of any viable cells as indicated by standard histological techniques of hematoxylin and eosin staining (Fig. 2B, C). No intact nuclei are observed in decellularized lungs compared to the control native lung (Fig. 2A). Some deep blue remnants are occasionally observed in vessel-mediated decellularized lung (Fig. 2B) indicating incomplete removal of nuclear debris. In contrast, the airway-mediated decellularized lung (Fig. 2C) shows no blue staining remnants or nuclear debris.

EVG staining was used to evaluate elastic fibers. Elastin staining in the control native lung appears as dark wavy fibers (Fig. 2D). The intensity of the stain decreases in the airway group (Fig. 2F) more so than in the vascular group (Fig. 2E). Alcian blue histologic staining was used to evaluate the amount of GAGs. GAGs were noticeably diminished in decellularized tissues (Fig. 2H, I) compared to native lung (Fig. 2G).

### Protein expressions

**FIG. 3.**
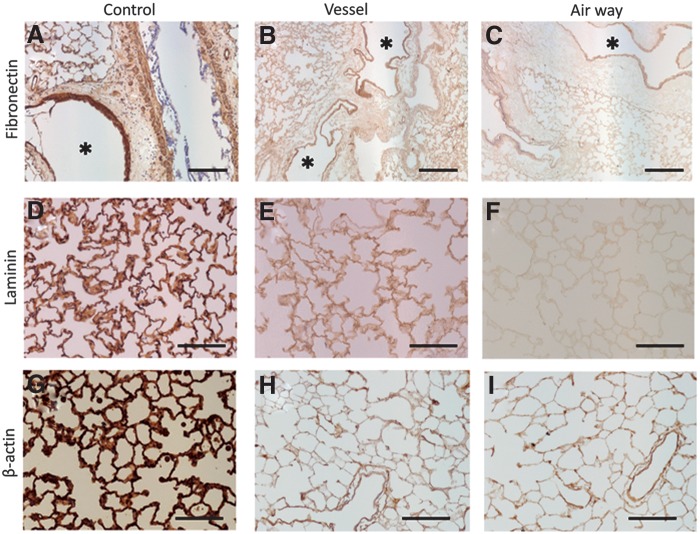
Immunostaining. Upper panel shows immunostaining for fibronectin in decellularized lungs **(B, C).** Both alveolar septa and large vessels (*) are strongly positive in native lung **(A).** The staining is weaker in vessel-mediated decellularized lung and airway-mediated decellularized lung **(B, C)**. The middle panel shows immunostaining for laminin in decellularized lungs **(E, F)**. Alveolar septa are strongly positive in native lung **(D)**. The staining is weaker in vessel-mediated decellularized lung and airway-mediated decellularized lung **(E, F)**. The lower panel shows immunostaining for β-actin in decellularized lungs **(H, I)**. Alveolar septa and pleura are strongly positive for β-actin in native lung **(G)**. The staining is weaker in vessel-mediated decellularized lung and airway-mediated decellularized lung **(H, I)**. Scale bar = 200 μm **(A–C)**. Scale bar = 100 μm **(D–I)**.

Immunohistochemical staining of fibronectin reveals that large vessels were immunopositive for fibronectin (Fig. 3A–C). The alveolar septa were immunopositive in both decellularized groups, but weaker in the airway group (Fig. 3C). Similarly, laminin was positive for alveolar septa in the vessel group, but weakly positive in the airway group (Fig. 3E, F). The immunostaining of β-actin, which is a component of the cellular cytoskeleton, showed that cell components were largely eliminated at the alveolar septa in both decellularized groups (Fig. 3H, I).

**FIG. 4.**
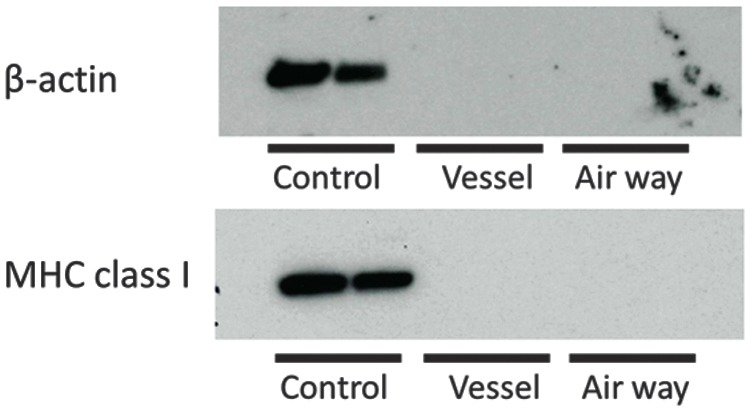
Western blot analysis for β-actin and MHC class I in decellularized lungs. The cell components of β-actin and cell membrane protein of MHC class I are not detected in both decellularized groups.

Western blot analysis shows that MHC class I was not detected in both decellularized groups (Fig. 4). β-actin was also not detected in decellularized groups, suggesting cell components were completely eliminated regardless of the decellularization method (Fig. 4).

### Collagen, GAG, and DNA contents

**FIG. 5.**
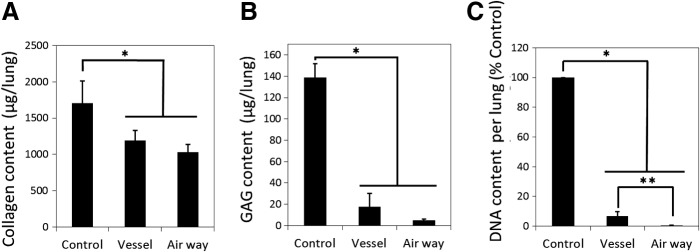
Collagen **(A)** sulfated GAG **(B)** and DNA **(C)** quantification of native and decellularized lungs. Asterisk indicates significance of difference between the groups (*p < 0.005, **p < 0.05). Data are expressed as mean value ± standard deviation, *n* = 5.

Collagen assay showed that collagen content was significantly decreased in both decellularized lung groups compared to the control group (*p* < 0.02), but not statistically different between the two decellularization lung groups (Fig. 5A). The collagen content in decellularized lungs was greater than 70% of the native control lung.

The GAG content of the decellularized scaffolds was significantly lower compared with native lung (*p* < 0.005). The GAG content in the vessel group was 12.5% of the native lung level and the airway group was 3.3% that of the level of the native lung (Fig. 5B).

The DNA assay demonstrated that the DNA content of the decellularized scaffolds was significantly lower compared with native lung (*p* < 0.00001) (Fig. 5C). The DNA content was significantly lower in the airway group than in the vessel group (*p* < 0.02).

### Scanning electron microscopy

**FIG. 6.**
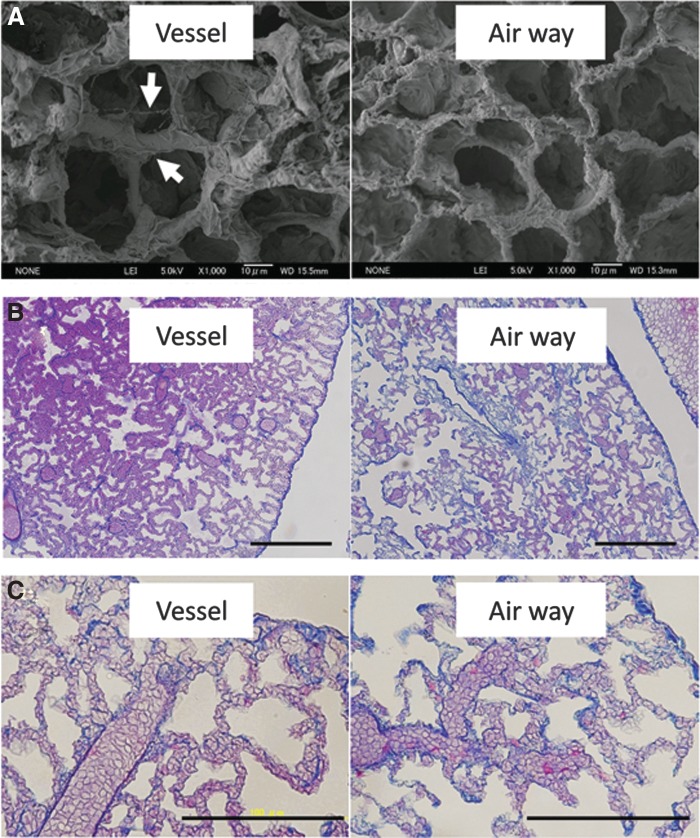
**(A)** Scanning electron microscopy (SEM) confirmed that alveolar ducts and alveolar network were completely preserved with an absence of cell bodies in both decellularized groups. The septa of alveoli in airway-mediated decellularized lung are thinner than those of vessel-mediated decellularized lung. **(B, C)** Red blood cell administration test showed that red blood cells were lined in the alveolar septa in both decellularized groups. Scale bar = 200 μm **(B)**. Scale bar = 100 μm **(C)**.

Scanning electron microscopy (SEM) confirmed that alveolar ducts and alveolar network were completely preserved with an absence of cell bodies in both decellularized groups (Fig. 6A). It is apparent that the septa of alveoli in the airway-mediated decellularized lung are thinner than those of the vessel-mediated decellularized lung. Wavy residue was not observed in the airway group (Fig. 6A; white arrows).

### Red blood cell administration test

To test the physical integrity of the decellularized alveolar capillaries, permeability was evaluated after red blood cell administration through the pulmonary artery. In the Masson's trichrome stain, red blood cells did not leak from the alveolar septa to alveolar spaces both in the vascular and the airway group, suggesting that capillary walls in the alveolar septa were preserved during both decellularization procedures. If there were any basement membrane ruptures of microcapillaries, they were smaller than 8 μm, which is the size of human red blood cells (Fig. 6B, C).

## Discussion

The present study was undertaken to improve the decellularization of lungs for possible use in organ transplantation. We investigated the decellularization efficacy and ECM preservation in airway-mediated decellularized scaffolds compared to ordinary vessel-mediated decellularized scaffolds. In a previous study, we used negative pressure ventilation in the timing of cell seeding of the decellularized rat lung. However, to our knowledge, airway-mediated decellularization using negative pressure with PEEP ventilation is a novel method for decellularizing the lung. Based on our histological and biochemical observations, using negative pressure for infusion has the advantage of achieving uniform decellularization.

Ventilation-based decellularization has a technical advantage over vessel-mediated decellularization or other airway-mediated decellularization. Vessel decellularization requires frequent drainage of used detergent or PBS, and drainage time cannot be calculated because that is determined by the patency of the vasculature (i.e., lack of thrombus). Price et al. reported another airway decellularization method in a mouse model.^10^ However, they repeatedly perfused the lung by injecting solutions into the trachea with a syringe, whereupon the solutions were expelled spontaneously due to recoil of the lung after removing the syringe. They also reported automated decellularization in human-sized lungs through airway and vasculature. In that system, detergents were pushed in and aspirated out from the trachea using a peristaltic pump.^11^ Compared with such a system, a ventilation-based decellularization using negative pressure in the bioreactor is more compatible with the physiology of the lung. And the ventilation system can also drain detergents and wash solutions from the lung into the waste bottle automatically. Therefore, we can calculate the decellularization time by the tidal volume and frequency of ventilation.

Our results show that airway-mediated ventilation-based decellularization results in a uniform decellularized scaffold of the lung. Histologically, the alveoli of the airway decellularization group were thinner than those of the vessel decellularization group on SEM. In addition, nuclear debris was minimal in the airway decellularized lungs compared to the vessel decellularized lungs. The reason for the efficiency of the airway decellularization might be due to the difference in function between airway and vessel systems. The ordinary vessel decellularization uses positive pressure of 20 cmH_2_O. Vessel decellularization solution starts from the pulmonary artery at the central part of the lung, moves through the microcapillary of the alveoli, with drainage occurring from the pulmonary vein, but with a large amount of drainage occurring through the airway. Therefore, insufficient decellularization might occur at the peripheral regions of the lung. In contrast, airway decellularization uses negative pressure in a closed circuit. Therefore, similar to physiological conditions, the alveoli begin to open at the periphery of the lung and decellularization solution starts at the subpleural area and spreads to the rest of the lung. In addition, the gap of the detergent surface level creates PEEP, which adds 11–15 cmH_2_O positive pressure in the alveolar space during the decellularization process and prevents collapse of the alveoli.

A disadvantage to airway-mediated decellularization is that the ECM content was reduced in the airway decellularized lungs compared to the vessel decellularized lungs. Staining with EVG and alcian blue revealed that elastic fibers and proteoglycans were both decreased by airway-mediated decellularization. The immunostaining of fibronectin and laminin clearly showed that both proteins were decreased in decellularized tissues, especially in airway-mediated decellularized lung. Elastic fibers endow vascular and pulmonary tissues with resilience and are critical to the function of arteries,^12^ allowing for intrinsic recoil properties.^13^ GAGs help to control macromolecular and cellular movement across the basal lamina,^14,15^ and GAGs on proteoglycans also affect the stretching and folding pattern of the fibers.^16^ Fibronectin and laminin have a prominent role in the formation and maintenance of vascular structures and are also a complex adhesion protein found in the ECM, especially within the basement membrane.^17^

However, the collagen content in decellularized lung was only reduced to about 70% of the native lung and was not statistically significant between vessel- and airway-mediated decellularization. In addition, alveolar hemorrhage was not observed with either decellularization method after red blood cell administration. Furthermore, DNA content was almost completely removed from the airway-mediated decellularized lung. Because DNA can be a strong immunoactivator, airway-mediated decellularization might have an advantage in producing a nonimmune organ compared to vessel-mediated decellularization. It is possible that other detergents, detergent concentrations, and wash volumes may result in less ECM being removed while retaining efficiency of the system for removing DNA. Further experiments with decellularization conditions and ultimately recellularization and transplantation will be needed to assess functionality of the engineered lung.

## Conclusion

Airway decellularization using negative pressure with PEEP ventilation is a novel method for decellularization of the lung. Our comparison of two methods of decellularization clearly shows that airway-mediated decellularization is more efficient for decellularization. The airway-mediated decellularization process reduces GAG, elastic fibers, proteoglycans, and laminin, but not collagen, compared to vessel-mediated decellularization. Although the matrix strength and clinical performance of the lung are unknown and further investigation is needed, airway-mediated ventilation-based decellularization provides another option in the field of whole organ decellularization.

## Abbreviations Used

DAPI: 4′, 6-diamidino-2-phenylindole
ECM: extra cellular matrix
EVG: Elastic Van Gieson
GAG: glycosaminoglycan
PEEP: positive end-expiratory pressure
SEM: scanning electron microscopy
